# Beneficial role of student societies in dermatology education

**DOI:** 10.1111/ajd.13820

**Published:** 2022-03-21

**Authors:** Sean E. Mangion, Chon‐Wai J. Chan, William Luu

**Affiliations:** ^1^ Sydney Medical School University of Sydney Camperdown New South Wales Australia; ^2^ UniSA: Clinical and Health Sciences University of South Australia Adelaide South Australia Australia; ^3^ Therapeutics Research Centre Basil Hetzel Institute for Translational Health Research The Queen Elizabeth Hospital Woodville South Australia Australia

**Keywords:** dermatology teaching, interest groups, learning activities, medical education, medical students.

Dear Editor,

Student societies form an integral part of the medical education experience. They include national‐level organisations (*e.g*. the Australian Medical Student Association, AMSA), state‐level collaborations (*e.g*. the New South Wales Medical Students' Council, NSWMSC) and local university‐based medical societies and special interest groups. Together, they provide student advocacy, educational resources, tutelage, as well as networking and social opportunities. To our knowledge, there are no reports about the role of these societies in dermatology education in Australia. Here, we summarise the current benefits offered by student dermatology societies, outlining the challenges and opportunities they face and highlighting how we can learn from student groups abroad to maximise their value.

Several dermatology societies operate in Australia and New Zealand, with benefits that range from additional lecture material to discounted training courses and scholarships (Table 1). These societies bridge the gap between content and skills that may be overlooked in the core medical curricula, which is critically important given the variability in teaching reported.^1^ Learning activities can be broadly classified into 5 categories^2^: (1) lectures/revision workshops focused on theoretical concepts; (2) clinical seminars with case presentations to place theory into practice; (3) journal clubs to critically appraise research; (4) online clinical modules to develop procedural skills (*e.g*. dermoscopy); and (5) bioethics training to better equip students to sensitively handle ethical dilemmas. It should be noted that these cover areas that the Australasian College of Dermatologists (ACD) has also recognised as priorities in its roadmap to improving patient care.^3^

**Table 1:** Characteristics of dermatology societies in Australia and New Zealand

Society	Target members	Cost	Member benefits
Sydney University Dermatology Society	Australian medical students	Free	Educational materials, annual Sydney Medical Students' Skin Conference, discounts to skin cancer courses and medical equipment, scholarships
Melbourne University Dermatology Interest Group		Free	Seminars, spot diagnosis competitions with gift vouchers
University of New South Wales Dermatology Society		Free	Revision tutorials, annual Sydney Medical Students' Skin Conference
University of Adelaide Skin Society		Free	Revision tutorials, educational resources
University of Queensland Dermatology		Free	Academic peer tutoring, summary lectures for examinations, research journal clubs
University of Newcastle Dermatology Society		$2	Practice questions, dermatology notes, competition series with gift vouchers
Flinders Society for Dermatology		Free	Monthly research and case discussions (Last Facebook activity on 22/02/15)
Dermatology Interest Group – Auckland	Auckland medical students	Free	Dermatology ward rounds, topic talks and national conference

A key challenge facing dermatology societies in Australia is that few exist and most operate in isolation. At the same time, they have the problem of encouraging adequate student involvement compared with other interest groups such as surgery or general practice. This might be due to the perception of dermatology as a niche speciality,^4^ coupled with the low availability of training positions and lack of exposure students receive in the core curriculum. A contributing factor to these challenges is that due to the shortage of dermatologists in Australia, medical students must compete with interns, residents and registrars for consultant teaching time.

Our experience at the Sydney University Dermatology Society has taught us the importance of collaborating to succeed against these challenges. Over the past year, we have joined forces with societies in New South Wales and interstate to share the task of hosting topic talks in diverse areas such as skin cancer, hidradenitis suppurativa, the skin microbiome, skin of colour, skin biopsy techniques, cutaneous infectious diseases and dermatologic emergencies. We also co‐hosted the Sydney Medical Students’ Skin Conference with further talks and workshops in ultrasound diagnostics and suturing. Partnering with societies in adjacent disciplines such as pathology, general practice and pharmacy has also allowed us to increase student involvement.

While the COVID‐19 pandemic has disrupted in‐person events, it has opened the opportunity of leveraging remote teaching and learning. For instance, using social media live streaming we saw engagement increase nearly 20‐fold (from an average of 30 participants to over 900 viewers using Facebook Live). Social media metadata can also provide insights into the characteristics of students interested in dermatology. At present, 57% of our page subscribers are female, with 55% 18–24 in age and 37% 25–34. This information may be useful in understanding changing demographics over time and may allow targeted dissemination of resources for prospective training programme candidates.

**Figure 1:**
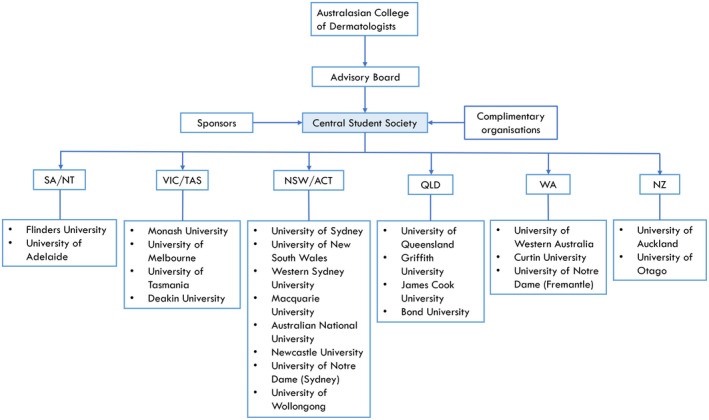
Proposed model for a national dermatology student society in Australia. [Colour figure can be viewed at wileyonlinelibrary.com]

To further improve dermatology societies, we can learn from similar groups operating overseas, particularly in the United States (US)^2^, ^5^, ^6^ and the United Kingdom (UK).^7^ The US has the Dermatology Interest Group Association (DIGA), and the UK has the National DermSoc Committee, supported by the British Association of Dermatologists, both of which act as centralised organisations working towards equal access to benefits for students. A similar model could be adopted here in Australia (Fig. 1), with endorsement by the ACD, and would have the advantage of maximising the use of limited resources (*e.g*. clinician time, equipment and funds). It would also provide a larger platform for networking, greater opportunity for advocacy, especially for minority groups such as Indigenous or LGBTQI+ students, and outreach to the broader community (*e.g*. DIGA has run sun safety awareness days where students are involved in assisting with community skin checks and educating about skin cancer prevention^8^).

Furthermore, a national‐level student society could enable active feedback about content in the medical curriculum and may open the door for the co‐creation of a standardised framework or guidance on teaching, similar to efforts in the UK.^9^ This is especially important given dermatology encompasses some 4000 individual skin, hair and nail conditions,^10^ and several difficult questions therefore arise for the educator: what should I teach? What have students learnt previously? What do they need and want to learn? Having student representation from individual schools will provide an inside voice that may help to (1) better define priority areas for teaching, (2) encourage its adoption and (3) provide insights into teaching barriers that may exist. Lastly, a national society may also serve as a central location for information about research opportunities and electives, which can extend not only to medical schools but hospitals and junior doctors who may wish to enter the dermatology training programme, in this way providing an opportunity to gain valuable mentorship and/or teaching experience.

More can be done in Australia to improve the exposure of medical students to dermatology. We propose that a national collaboration run by students and backed by the ACD is an ideal solution to this problem.
